# Micro-Raman Spectroscopy of Silver Nanoparticle Induced Stress on Optically-Trapped Stem Cells

**DOI:** 10.1371/journal.pone.0035075

**Published:** 2012-04-13

**Authors:** Aseefhali Bankapur, R. Sagar Krishnamurthy, Elsa Zachariah, Chidangil Santhosh, Basavaraj Chougule, Bhavishna Praveen, Manna Valiathan, Deepak Mathur

**Affiliations:** 1 Centre for Atomic and Molecular Physics, Manipal University, Manipal, India; 2 Stempeutics Research Pvt. Ltd., Manipal, India; 3 Department of Pathology, Kasturba Medical College, Manipal, India; 4 Tata Institute of Fundamental Research, Mumbai, India; Joint Research Centre - European Commission, Germany

## Abstract

We report here results of a single-cell Raman spectroscopy study of stress effects induced by silver nanoparticles in human mesenchymal stem cells (hMSCs). A high-sensitivity, high-resolution Raman Tweezers set-up has been used to monitor nanoparticle-induced biochemical changes in optically-trapped single cells. Our micro-Raman spectroscopic study reveals that hMSCs treated with silver nanoparticles undergo oxidative stress at doping levels in excess of 2 µg/ml, with results of a statistical analysis of Raman spectra suggesting that the induced stress becomes more dominant at nanoparticle concentration levels above 3 µg/ml.

## Introduction

There has been a rapid growth of applications of nanotechnology to diverse areas of human endeavor, with considerable interest in potential uses of nanoparticles in diverse physical and biological systems. Recent work seems to offer indications that, despite a plethora of potential benefits, nanoparticles (NPs) may also generate adverse effects that are a consequence of their size-related properties. The very considerable attention focused on beneficial effects of NPs, such as their antibacterial [1]–[5], antiviral [6] and antitumor [7] properties, has not always been matched by thorough investigations of possible health hazards at the cellular, molecular, or whole organismal level in eukaryotes, even though there is clinical and experimental evidence to suggest that the small size of NPs coupled with their large surface area, along with the ability to generate reactive oxygen species, contributes to their potential to induce cell injury [8]. It is the purpose of our investigation to probe concentration-dependent biochemical changes that may be induced in single, live cells by silver nanoparticles (Ag NPs); such biochemical changes may correlate to changes in cell morphology, surface marker expression and cell proliferation and viability.

We have chosen to utilize human mesenchymal stem cells (hMSCs) as an experimental in-vitro model for our studies. The experimental tool that we utilize is single-cell Raman spectroscopy that is implemented in a Raman Tweezers set-up that enables individual live cells to be optically trapped by means of a very low power laser beam. The wavelength (1064 nm) of our laser light neither causes photodamage nor induces photochemical changes within live cells. Raman fingerprints of biological materials offer deep insights into physiochemical properties and, indeed, Raman spectroscopy coupled to optical tweezers has begun to be utilized in recent years for studies involving red blood cells [9]–[11].

Optical tweezers have proved to be of widespread utility in contemporary research in the biomedical sciences [12], [13]. Tweezers rely on the use of tightly-focused laser light to create a sharp gradient of light intensity over very small spatial dimensions such that microscopic dielectric objects floating in liquid media in the proximity of the laser focal volume are trapped by the action of a gradient force [14]. Such trapped objects can then be readily probed using highly-sensitive spectroscopic methods [15]–[33] in such manner that the deleterious effects on spectral quality of the inevitable Brownian motion are successfully circumvented. Combination of spectroscopy with optical tweezers thus opens new experimental vistas for extraction of precise information about biochemical changes at a single-cell level under physiological conditions and without the necessity to chemically fix cells of interest. This combination constitutes an important advantage as cell immobilization by chemical or physical means may often lead to alteration of the physiochemical microenvironment which may, in turn, result in changes in electrochemical potentials across the cell membrane such that cellular functions are affected [16]. From the viewpoint of probing biochemical changes that occur during the transformation of cells from their normal stage to some abnormal stage (disease, or externally-induced abnormalities such as chemical stress and nanoparticle induced stress) it is clearly desirable to use an experimental technique that ensures that results are not influenced by factors such as cell death or oxidative stress that may be induced by chemical fixing procedures.

In order to facilitate spectroscopic studies of single living cells in a physiological medium, we have developed a high-resolution, dual-wavelength apparatus that combines optical trapping with Raman spectroscopy, utilizing near infrared wavelength light at 1064 nm for trapping and 785 nm light for Raman excitation at very low levels of incident power (<10 mW). Among several spectroscopy techniques, Raman spectroscopy is a particularly potent tool to probe the biochemical composition of cells; it has proved to have the potential of being able to differentiate between, for example, normal and malignant cells [20]–[22]. In recent years, micro-Raman spectroscopy has developed into a powerful tool to spectroscopically probe single cells with high spatial resolution, requiring relatively simple sample preparation procedures [34]–[38]. Resonance Raman spectroscopy has also been utilized to probe DNA bases and aromatic amino acids in proteins, and the technique has been successfully applied to rapidly identify bacteria such as E. coli, P. fluorescens, S. epidemis, B. subtilis and E. cloaca [37], [38]. We note that even micro-Raman methods require the sample cells to be adsorbed on to a microscope coverslip, making them chemically far removed from physiological conditions.

The relatively recent confluence of optical tweezers and Raman spectroscopy [15], giving rise to Raman Tweezers, has already resulted in a number of interesting and important biomedical applications [15]–[33]. Raman Tweezers have begun to facilitate the acquisition of spectroscopic information with good spatial resolution and identification of intra-cellular components under physiological conditions. In early work, Raman Tweezers comprised a single, low power laser beam, usually of 785 nm wavelength, that served the dual purpose of optical trapping and near-infrared Raman spectroscopy [15]. The advantages of using a dual wavelength set-up for confocal Raman spectroscopy have been established by Petrov and coworkers [17]; one of the two laser beams was used by them for trapping micron-size cells and a second laser beam was used to carry out Raman excitation. As described below, it is the dual beam method that we utilize in our present investigations.

Stem cell therapy is a rapidly evolving area of research in regenerative medicine [39]–[40]. Mesenchymal stem cells are neither transformed cell lines nor immortalized cells but represent primary cells that can be cultured over several passages. Furthermore, hMSC's are found in different tissues such as bone marrow, fat, or muscle [41]. This cell type is intimately involved in tissue regeneration and repair [42], [43] because of its ability to differentiate into various tissues of mesenchymal origin (bone, cartilage, fat, muscle, marrow stroma, tendon, ligament, and other connective tissues) [44]. The connection between NPs and stem cells arises from the use of the former in contemporary schemes for overcoming persistent technical challenges in therapeutic applications [45]. Despite the wide range of applications of NPs in the stem cell field, there continues to be a paucity of information concerning the impact of nanomaterials on human health and the environment [46]. Biocompatibility of nanoparticles is, obviously, the most important prerequisite for their applications in biomedicine, but questions remain as to what criteria are to be adopted to evaluate their potential toxicity [47]. NP toxicity is of particular importance for stem cells where their effect on the potential for self-renewal and differentiation remains unknown.

Data on NP toxicity in stem cells continues to remain scarce. The molecular mechanisms of NP toxicity in general are still poorly understood [46] although there are suggestions that oxidative stress and lipid peroxidation (LPO) may play an important role in NP-elicited DNA damage, cell membrane disruption and subsequent cell death [48]–[61]. As far as silver NPs are concerned, they have achieved the highest degree of commercialization among nanomaterials [62]. Silver NPs have well known antimicrobial properties, and are now extensively used in diverse industrial applications from clothes and catheters to electrical home appliances and biomedical implants. However, despite their widespread use, there is a dearth of information on the biological effects of Ag NPs on human cells.

In the experiments that we report here, we have sought to probe the effect that different concentrations of silver NPs have on hMSCs. Established biotechnological assays to probe NP cytotoxicity have proved to be time consuming and they are, quite often, limited in their capacity to elucidate underlying biochemical mechanisms [63]. We chose to apply Raman spectroscopy to single cells that are optically trapped under physiological conditions as an alternative technique to assess the cellular response that is induced upon NP exposure. In recent work, the combination of optical tweezers and Raman spectroscopy has provided us with a robust platform to probe biochemical changes experienced by single cells due to external chemical stress agents [64]. Our experiments allow us to deduce that Ag NP concentration levels as low as 1 µg/mL induce oxidative stress in cells. In order to test the severity of the concentration dependent modifications on cells, we have carried out statistical analysis (using Principal Component Analysis - PCA) on a number of cells; PCA results show a clustering of data points (score of factor 1) for cells treated with NP concentrations less than 3 µg/mL, whereas at concentration levels in excess of 3 µg/mL the PCA results are more scattered.

## Methods

### Ethics Statement

Ethical clearance (reference SPRL/CLI/07-08/001, dated 14/02/2008) was obtained from the Manipal University Ethics Committee to collect bone marrow aspirations from healthy volunteers to isolate and culture bone marrow derived adult allogenic mesenchymal stem cells for the purposes of the research reported in this paper. Informed written consent was obtained from all volunteers.

### Nanoparticle Preparation

The Ag NPs used in the present study were procured from Sigma Aldrich. The average size of the nanoparticles was 100 nm; they were dispersed in sterile, ultra-pure water at 1 mg/ml as stock solution and final concentrations of 1, 2, 3, 4 and 5 µg/ml were prepared by serial dilution of 1 ml stock solution in sterile ultra-pure water. Since our focus of study is single-cell Raman spectroscopy of hMSCs and oxidative stress induced by Ag NPs on hMSCs, as described in the following, control experiments were carried out on stem cells under the same conditions but without nanoparticles. Since the concentrations of nanoparticles taken for incubation with cells were very low, collection and characterization of the NPs after the incubation was not possible. However, we do not expect any chemical and physical changes on the Ag NPs due to the presence of stem cells.

### Processing of human MSCs

The bone marrow aspirate collected was diluted with complete media (KO-DMEM, GIBCO 10829 Invitrogen, Auckland) containing 10% Fetal Bovine Serum (Cat No. SH 30084.03, Australia), Hyclone lot selected for rapid growth of human BM-MSC's, 1% L-Glutamine (Sigma G7513, USA) and 0.5% Penicillin-Streptomycin (GIBCO 15140, Invitrogen, Auckland) in the ratio of 1∶2 was gently mixed and filtered through a 100 µm cell strainer (BD Falcon, 100 µm Nylon, REF 352360, USA). The suspension was centrifuged (Hettich Rotina 420R) at 1200 rpm for 10 minutes at room temperature. The pellet obtained was dissolved with complete media and gently overlaid on to the lymphoprep (Axis shield, lymphoprep, 07K13S06) in the ratio of 1∶2, preventing formation of a homogenous mixture; the combination was centrifuged at 1800 rpm for 10 minutes at room temperature. The buffy layer containing mononuclear cells enriched with MSCs present at the interface was carefully aspirated and collected in a centrifuge tube; equal volume of complete media was added and centrifuged at 1200 RPM for 10 minutes at room temperature.

**Figure 1:**
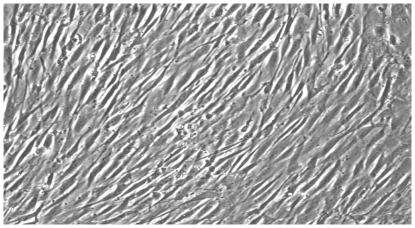
Phase contrast microscope image of control human Mesenchymal stem cells (hMSC) culture after passage 5. Magnification 4×.

**Figure 2:**
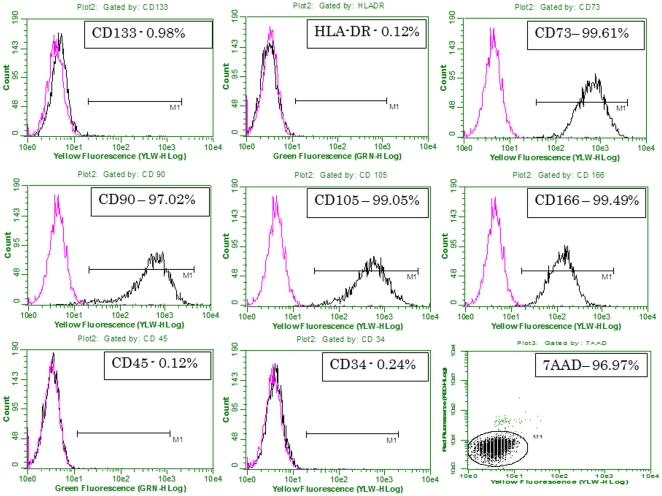
Flow cytometer CD Marker Expression for control hMSC (without Ag NPs).

The obtained pellet was homogenized with 5 ml of complete media and was gently re-suspended in a culture flask and incubated at 37°C with 5% humidified CO_2_ (Thermo Scientific Hera cell 240 series). After 4–5 days of incubation, the culture flask was checked for adherent cell colonies/population and the media was replenished. This procedure was continued until 70%–80% confluency was attained and the flask's contents were further subjected to trypsinisation. The cells were characterized by monitoring the cell morphology using phase contrast microscopy (Fig. 1) as well as Flow Cytometry CD marker expression essay (Fig. 2).

Four-color cytometry was performed using a Gauva Easycyte Plus set-up. After trypsine treatment and centrifugation at 1200 rpm for 10 minutes, the cells were prepared for flow cytometric analysis. Typically one million cells were resuspended in 1000 µl of PBS and 100 µl of uniform cell suspension containing 1×105 cells was distributed into a well of a non-adherent 96 well plate (Micro Test Plate: Tarsons 941296). 5 µl of respective antibody was added to each well including Iso PE and Iso FITC. The complete mix was incubated for 30 minutes at 4°C before analysis. The following anti-human antibodies were used: FITC (mouse IgG1 Cat. No. 550616 BD Pharmingen, BD Bioscience) conjugated antibodies to CD45 (Cat. No. 555482, BD Pharmingen, BD Bioscience), PE mouse Anti-human CD34 (Cat. No. 550761, BD Pharmingen, BD Bioscience), Anti-HLA-DR (Cat. No. 347363 BD Bioscience) PE (mouse IgG1 Cat. No. 550617 BD Pharmingen, BD Bioscience) conjugated antibodies, antihuman CD44 (Cat. No. 550989, BD Pharmingen, BD Bioscience), PE Anti human CD73 (Cat. No. 550257, BD Pharmingen, BD Bioscience), PE Anti human CD90 (Cat. No. 555596, BD Pharmingen, BD Bioscience), PE Mouse IgG1 CD105 (Cat. No. FAB10971P, R&D Systems) and PE mouse anti-human CD166 (Cat. No. 559263, BD Pharmingen, BD Bioscience). After incubation, fluorescence measurements were carried out. The viability of the cells was analyzed by 7AAD (Cat. No. 51-68981E, BD Pharmingen, BD Bioscience) using Gauva Easycyte Plus and was evaluated with Cytosoft 5.2.

Surface antigens were detected by flow cytometery for hMSC's. Figure 2 shows our flow cytometric analysis hMSC's at passage 5. The antigens tested were CD45, CD34, CD133, HLA-DR, CD73, CD90, CD105 and CD166. The hMSC's were positive for CD73, CD90, CD105 and CD166 and negative for CD45, CD34, CD133 and HLA-DR. The viability of hMSC's for 7AAD was above 90% as shown in Fig. 2.

The bone marrow aspirate collected was diluted with complete media (KO-DMEM, GIBCO 10829 Invitrogen, Auckland) containing 10% Fetal Bovine Serum (Cat No. SH 30084.03, Australia), Hyclone lot selected for rapid growth of human BM-MSC's, 1% L-Glutamine (Sigma G7513, USA) and 0.5% Penicillin-Streptomycin (GIBCO 15140, Invitrogen, Auckland) at a ratio of 1∶2 was gently mixed and filtered through a cell strainer of 100 µm (BD Falcon, 100 µm Nylon, REF 352360, USA). The suspension was centrifuged (Hettich Rotina 420R) at 1200 RPM for 10 minutes at room temperature. The pellet obtained was dissolved with complete media and gently overlaid on to the lymphoprep (Axis shield, lymphoprep, 07K13S06) at a ratio of 1∶2, preventing a homogenous mixture formation; the combination was centrifuged at 1800 RPM for 10 minutes at room temperature. The buffy layer containing mononuclear cell enriched with MSCs present at the interface was carefully aspirated and collected in a centrifuge tube; an equal volume of complete media was added, followed by centrifugation at 1200 RPM for 10 minutes at room temperature.

The obtained pellet was homogenized with 5 ml of complete media and was gently re-suspended in to a culture flask and incubated at 37°C with 5% humidified CO_2_ (Thermo Scientific Hera cell 240 series). After 4–5 days of incubation, the culture flask was checked for adherent cell colonies/population and the media was replenished. The same was continued until 70%–80% confluency was attained and further subjected for trypsinisation. The cells were characterized by monitoring their morphology using phase contrast microscopy (Figure 1) as well as using CD marker assay (Figure 2).

### Culture of hMSCs and incubation with nanoparticles

Bone marrow derived Mesenchymal stem cells were cultured in-vitro with complete media comprising KO-DMEM, GIBCO 10829, Invitrogen, Auckland containing 10% Fetal Bovine Serum (Cat. No. SH 30084.03, Australia), Hyclone - lot selected for rapid growth of human BM-MSC's, 1% L-Glutamine, Sigma G7513, and 0.5% Penicillin-Streptomycin, GIBCO 15140, and different concentrations of Ag NPs. The cells were maintained at 37°C with 5% CO_2_; media were changed every 72 hours. The hMSC's were sub-cultivated every 7–14 days, depending on cell proliferation for two passages (3^rd^ to 5^th^ passage). Adherent cells were washed with phosphate buffered saline solution (GIBCO, Invitrogen) and detached by the addition of 0.25% trypsin (GIBCO, Invitrogen) for 2 minutes at 37°C. Subsequently, the hMSC's were collected and washed twice with complete media. At the end of the 9^th^ day cells were harvested from exponentially growing cultures using trypsin (0.25%). Cell suspensions were prepared and then dispersed within 96 well micro-titer plates (3×10^4^ cells/100 µl/well). Triplicate wells were used for each determination. Plates were incubated at 37°C with 5% CO_2_ for 72 hours, after which time they were treated with 17 different concentrations (50 ng, 100 ng, 150 ng, 200 ng, 250 ng, 500 ng, 1 µg, 1.5 µg, 2 µg, 2.5 µg, 3 µg, 3.5 µg, 4 µg, 4.5 µg, 5 µg, 6 µg, 7 µg, 8 µg, 9 µg, and 10 µg) of Ag NPs and incubated at 37°C with 5% CO_2_ for 72 hours. The cells in our experiments were kept in DPBS (Dulbecco's phosphate buffered saline) so as to maintain physiologically-relevant values of pH, salinity, etc. to ensure intact live cells.

### Micro-Raman spectroscopy

**Figure 3:**
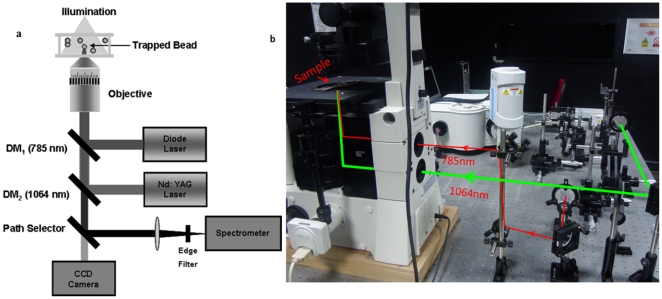
The experimental apparatus used in the present studies. (a) Schematic representation of the Raman Tweezers set-up. (b) Picture of the Raman Tweezers set-up showing the geometry of the trapping laser beam and the Raman probe laser beam.

The Raman Tweezers system (Figure 3) consists of an inverted microscope (Nikon Eclipse Ti-U, Japan) with a high numerical aperture (1.3 NA), 100× oil immersion objective (Nikon, Plan Fluor) to produce a diffraction limited focal spot to trap micro-particles suspended in solutions in a custom-made sample cell. An Nd∶YAG laser (Laser Quantum, UK) with an output beam of 1064 nm wavelength is used as the trapping laser. In order to overfill the back aperture of the objective, the laser beam cross-section is increased to nearly 9 mm diameter using a manual beam expander. The laser beam was the steered through a 1∶1 telescopic arrangement comprising two convex lenses of equal focal length f, kept at a distance of 2f from each other. A dichroic mirror having high reflectivity at 1064 nm wavelength placed inside the microscope body, directed the laser beam to the back aperture of our microscope objective. A CCD camera (Nikon DS-2MBW, Japan) was attached to one of the exit ports of the microscope, enabling visual monitoring of the sample plane. Raman spectroscopy was carried out using a Diode laser (Starbright Diode Laser, Torsana Laser Tech, Denmark) with an output beam of 785 nm wavelength which also passes through a different beam expander and 1∶1 telescopic arrangement before getting reflected from another dichroic mirror having high reflectivity at 785 nm wavelength placed inside the microscope body. The reflected beam enters the same objective as the 1064 nm beam and forms the focus at the sample plane. Our 1∶1 telescopic arrangements allowed us to locate the foci both lasers at the same point in the sample plane so as to enable simultaneous trapping and excitation.

Details of our Raman Tweezers apparatus have been published recently [65] but it is important to briefly comment on our methodology for acquiring Raman spectra. The choice of 785 nm as the wavelength of the exciting laser was made after taking into account the following considerations. The efficiency of Raman scattering is known to exhibit a λ^−4^ wavelength dependence; shorter wavelengths are, therefore, desirable. However, as indicated by the work of Neuman and coworkers [66], short wavelengths also result in increased likelihood of laser-induced photodamage. Use of longer wavelengths, on the other hand, allows the possibility of reducing fluorescence effects that would compete with the weak Raman signals that are anticipated in the type of work that we wish to undertake on our stem cell samples. However, longer wavelengths present inescapable technical problems that are linked to the efficiency of CCD-based photon detectors that are readily available at present. As discussed earlier [26], currently the best available detection efficiency (∼50%) is obtained over the wavelength range 600–800 nm, with sharp fall-off at longer wavelengths: the efficiency value at 1000 nm wavelength falls to ∼10% while at 1064 nm it is almost zero. Our choice of 785 nm as the Raman excitation wavelength is, therefore, an optimum utilitarian compromise.

We have considered the possibility of utilizing the 785 nm light for both trapping and excitation. In our experiments, however, cognizance has to be taken of the fact that the strength of the optical trap depends strongly on the quality of the laser beam. Diode lasers that are commercially available for Raman excitation have distinctly inferior beam quality to what is routinely obtained from the 1064 nm wavelength Nd∶YAG laser that we utilize for trapping. As use of the Raman laser for trapping would result in considerably less efficient trapping, we have chosen to use a two-laser system for our Raman Tweezers work.

As discussed in earlier measurements reported from our laboratory [65], alignment and calibration of the experimental apparatus is an important prerequisite for our measurements. We accomplished alignment and calibration by making use of polystyrene beads (average diameter 3 mm, obtained from Sigma Aldrich, USA). A bead, suspended in deionised water, was trapped (typically using a trapping power of 5 mW of 1064 nm light) and the Raman spectrum was recorded by exciting it with 785 nm laser light (typically using power levels of ∼10 mW). The optics of our Raman spectrometer were aligned so as to achieve maximum signal-to-noise ratio in measured spectra. A recent report [65] has presented typical Raman spectra of a trapped polystyrene bead that we measure with integration times of 2 s and 10 s. The morphology and details of these spectra are fully in accord with those reported in existing literature [15]. Typically, we recorded as many as 20 spectra so as to establish reproducibility on the wavelength axis of at least ±1 cm^−1^. We measured laser power levels used to trap the cells and to record Raman spectra by locating an integrating sphere coupled to a photodiode just after the microscope objective in our experimental set-up (Fig. 3).

Spectral resolution is another important experimental parameter that was quantified prior to any series of spectral measurements. We established typical resolution of our system to be of the order of 5 cm^−1^; this value was readily obtained by keeping the spectrometer slit width at 100 µm, and measuring the FWHM of the 997 cm^−1^ Raman line of the polystyrene bead spectrum.

We took care to ensure that laser power levels that were incident on the trapped cell were low enough to ensure cell viability. In all our experiments, measurements were made on each trapped cell for periods as long as ∼1 hour and, in the course of this time Raman spectra was recorded every 5–10 minutes. We ensured that recorded spectra were consistent during the entire stretch of each experimental run to allay concerns regarding cell viability. Optical trap work on live cells that has been conducted in our laboratory over several years (see [64], [65], and references therein) has provided evidence that optically-induced damage is unlikely to occur at incident laser power levels that are less than ∼30 mW in our system provided that the irradiated cells are kept in a physiologically relevant medium, which they were. The lowest possible incident power levels were utilized to obtain spectra of adequate signal-to-noise ration and resolution.

In experiments with hMSCs, a micropipette was used to transfer ∼150 µl of cell suspension in DPBS on to the sample cell located within the focal volume of our optical trap set-up (Fig. 3). The sample cell was made by attaching a glass microscope cover slip on to a metal plate with a 1 mm deep wedge (dimensions: 0.8 cm width and 1 cm length). The sample cell was mounted on a controllable *x-y* translation stage that was used to bring a single cell into the alignment with the laser focal spot (using 1064 nm laser light), resulting in the trapping of the cell (as established by moving the translation stage and observing that the cell remained fixed by the laser focal spot while other proximate cells move along with the translation stage). The trapped cell was then irradiated by a second laser beam (of wavelength 785 nm). Light scattered from the trapped cell was collected by the same microscope objective and optically guided into the Raman spectrometer (see Fig. 3). A CCD-based photon detector, as described above, detected the spectrometer output.

**Figure 4:**
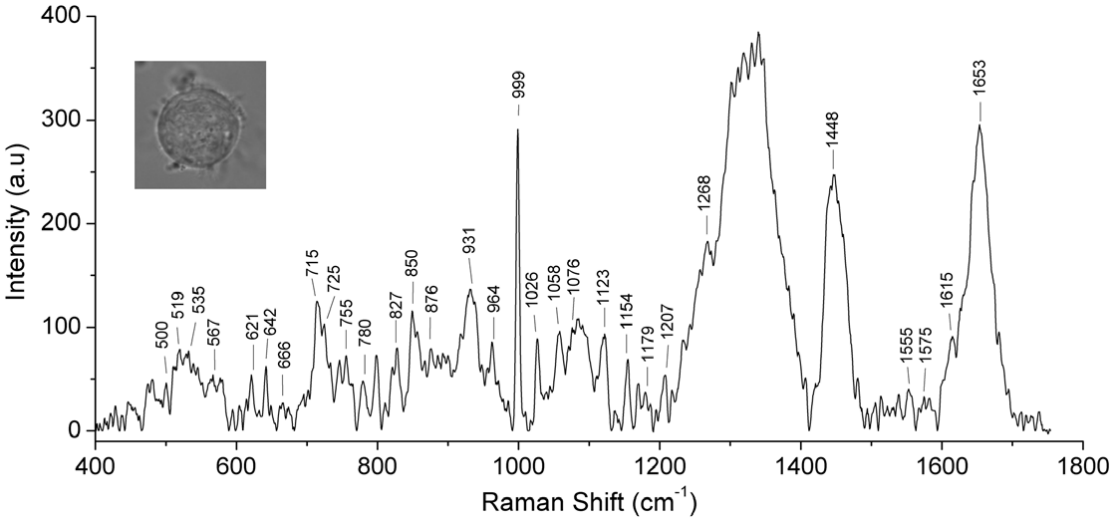
Morphology of hMSC's (A) Control, and Ag NP treated with concentration (B) 1 µg/ml, (C) 2 µg/ml, (D) 3 µg/ml, (E) 4 µg/ml and (F) 5 µg/ml. Magnification 4×.

Following this procedure, Raman spectra of control cells and NP-treated hMSCs were collected over the range 400–1800 cm^−1^, the spectral region known as the bio-molecular fingerprint region as it provides information about various functional groups of biological macromolecules of the cell under investigation. Raman spectra were not recorded from hMSCs treated with very low NP concentrations (50 ng–500 ng), because, we could not find any visible morphological changes in them. Figure 4 shows morphologies of hMSCs treated with Ag NPs (1–4 µgl) at passage 5. The changes in morphology becoming visible after 72 hours of exposure for cells treated with 1 µg/ml, 2 µg/ml, and 3 µg/ml respectively, with a few cells losing their spindle shape to become web shaped. At higher NP concentrations (4–5 µg/ml) cells became more stretched, losing their usual morphology becoming detached from the surface after 48 hours (see panel F) of NP incubation. All the above samples were further characterized by micro-Raman spectroscopy. Cells were completely disrupted at NP concentration >5 µg. Assignments of the various bands were made on the basis of available literature [67]–[73].

We have ensured that the concentration of nanoparticles used in our study was kept sufficiently low to preclude auto-oxidation induced by surface-enhanced Raman effects. This is made evident in the spectra we present in the following: there is no enhancement observed in the overall strength of the Raman signal that is obtained from our nanoparticle-treated cells. The absence of significant photochemistry induced by the 785 nm laser light was confirmed by recording Raman spectra of a given cell for 15 minutes at a time, with an interval of 1 minute between subsequent measurements. We ensured that all 15 spectra thus obtained retained the same Raman features, providing indication that no photoinduced damage was induced in the cell in the course of the experiment. We reiterate that extensive work has been carried out in our laboratory and by others (see the cogent review of such work by Snook and co-workers [26]) that establishes the lack of possible photo-induced effects initiated by 1064 nm laser light.

Raw spectra obtained in these experiments were subjected to 5-point moving averages smoothing and, subsequently, multi-point baseline correction was applied. Spectra were also measured of bare glass slides and these enabled us to identify certain spectral features as being contributed by the glass slide rather than the sample.

## Results and Discussion

Alignment and calibration of our experimental set-up was accomplished using polystyrene beads of average diameter 3 µm (Sigma Aldrich, USA). A bead suspended in deionised water was trapped, typically using a trapping power of ∼5 mW, and the Raman spectrum was recorded by exciting it with 785 nm laser light. Raman spectrometer optics were aligned for maximum signal-to-noise ratio in measured spectra. The spectral resolution of our system was determined to be ∼5.7 cm^−1^ (with the spectrometer slit width kept at 100 µm) by measuring the FWHM of the 997 cm^−1^ Raman band of the polystyrene bead spectrum.

**Figure 5:**
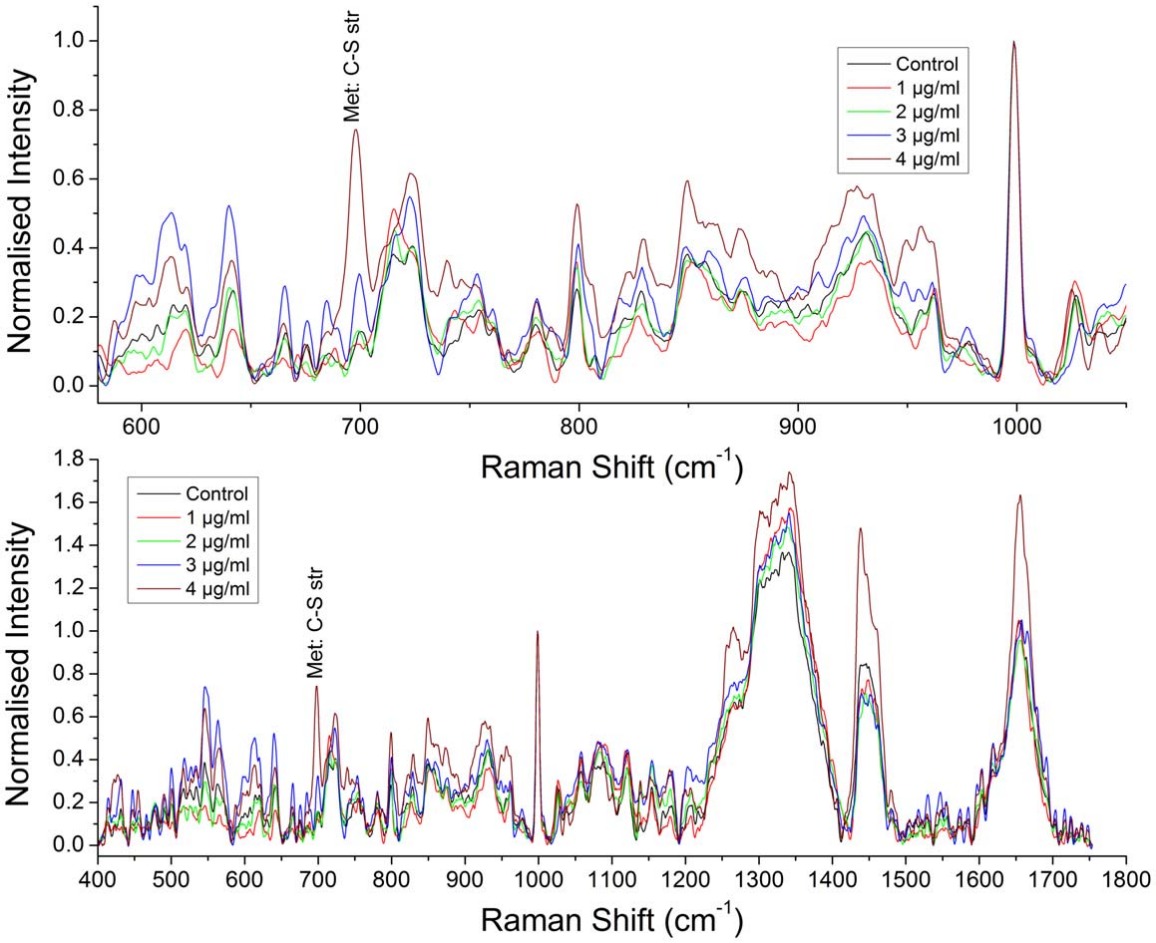
Shows a typical baseline-corrected microRaman spectrum of an optically trapped hMSC (an image of which is shown). The laser power used for trapping (1064 nm wavelength) was 5 mW while the Raman spectrum was measured using a laser power of ∼20 mW (785 nm wavelength); the acquisition time was 120 s and 5 accumulations were made.

Figure 5 depicts a typical Raman spectrum of a normal, live hMSC recorded using our Raman Tweezers setup; the peak assignments are tabulated in Table 1. The sizes of hMSCs used in these studies varied from 15 µm to 20 µm. The theoretical diffraction limited spatial resolution of our Raman Tweezers system (with 100× objective, NA of 1.3) was 0.36 µm. In similar fashion to recent results on live human red blood cells [65], it is clear from the spectrum shown in Fig. 5 as well as the contents of Table 1 that our spectrum of trapped hMSCs also yields rich information on Raman frequencies arising from vibrations of various proteins, lipids and polynucleotides. The prominent protein features include the Amide I band located at 1653 cm^−1^, the CH_2_/CH_3_ bending vibration at 1448 cm^−1^, Amide III in various conformations at 1268 cm^−1^ and 1207 cm^−1^, C-N stretching at 1026 cm^−1^, 1058 cm^−1^, 1090 cm^−1^, 1123 cm^−1^ and 1154 cm^−1^, and ring breathing vibrations of the phenylalanine molecule at 999 cm^−1^. We assign peaks at 500 cm^−1^, 519 cm^−1^ and 532 cm^−1^ to the protein S-S stretch for different values of dihedral angles of the CS-SC and SS-CC groups. The peaks at 642 cm^−1^ and 666 cm^−1^ originate from protein C-S stretching vibrations while C-S stretching in methionine is observed at 700 cm^−1^ [69]–[73].

**Table 1:** Raman frequency assignments of single control hMSC.

Raman Shift (in cm^−1^)	Assignments	Raman Shift (in cm^−1^)	Assignments
500	p^a^ :S-S str	964	p^a^: residue C-C str
519	p^a^ :S-S str	999	Phe^b^ : C-C skeletal
532	p^a^ :S-S str	1026	Phe^b^, p^a^:C-N str
567	Deoxyribose	1058	NA^f^ :C-O str, p^a^:C-N str
621	Phe^b^ : C-C twist	1076	Phospholipids: C-C Str
642	p^a^ : C-S str, Tyr^c^ : C-C twist	1090	DNA:bk:O-P-O sym str,P:C-N str
666	G,T, Tyr^c^, bk in RNA, p^a^ : C-S str	1123	p^a^ :C-N str
686	G	1154	DNA:RP, p:C-C, C-N str
700	Met^d^: C-S str	1179	Tyr^c^, Phe^b^, P:C-H bend
715	C	1207	Tyr^c^, Phe^b^, A, T, p:Amide III
725	A	1268	p^a^ :Amide III;C-H bend
755	Trp^e^	1302	A, phospholipid:CH2
780	C,T	1448	p^a^ :CH2 def
799	Artifact	1555	Trp^e^, p^a^ :Amide III
827	O-P-O asym str,Tyr	1575	G,A
850	Tyr^c^	1615	Tyr^c^, Trp^e^, p^a^:C = C
876	Trp^e^	1653	p^a^ :Amide I
931	p^a^ :bk C-C str		

a p:protein,

b Phe: phenylalanine,

c Tyr: tyrosine,

d Met: methionine,

e Trp: tryptophan,

f NA: Nucleic Acids.

A,U,G,T,C denote ring breathing modes of nucleic acid bases adenine, uracil, guanine, thymine, and cytosine.

The CH_2_ vibration and C-C stretching vibrations related to the lipids are responsible for the bands at 1302 cm^−1^ and 1076 cm^−1^, respectively. The bands located at 686 cm^−1^, 715 cm^−1^, 780 cm^−1^ and 1575 cm^−1^ are related to the vibrational modes associated with different DNA bases. The vibrations corresponding to DNA, O-P-O symmetric and asymmetric stretching, occur at 1090 cm^−1^ and 827 cm^−1^, respectively. It should be noted that there is a large background in the 1200–2000 cm^−1^ region that hides many characteristic peaks; results of systematic measurements made by us indicate that this background arises from the glass coverslip we used.

**Figure 6:**
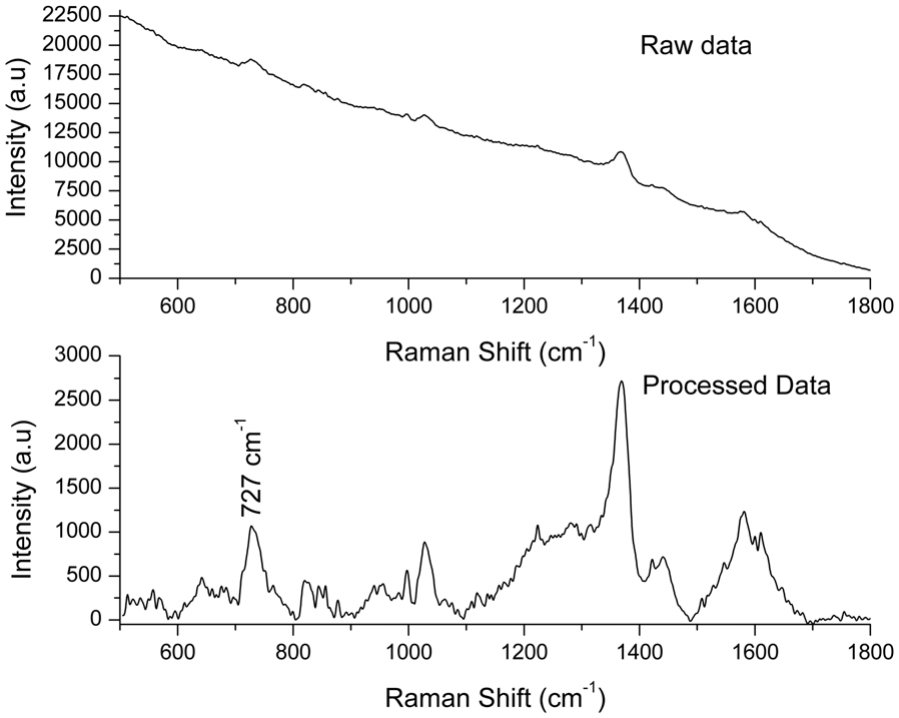
Raman spectra of hMSCs. Top panel shows normalized Raman spectra of hMSCs exposed to different concentrations of Ag nano-particles (1–4 µg/mL). The normalization was with respect to the 999 cm^−1^ peak. The laser power used for trapping (1064 nm wavelength) was 5 mW while the Raman spectrum was measured using a laser power of ∼20 mW (785 nm wavelength). Each spectrum is an average of spectra from five individual hMSCs. Acquisition time: 120 s and 5 accumulations. The lower panel shows the same spectra over an extended range (see text).

Raman spectroscopy of hMSCs treated with 1–4 µg/mL of Ag NPs was also performed and typical results are shown in Fig. 6. Exposure of hMSCs to higher concentrations (>4 µg/mL) of Ag NPs resulted in cell death. Comparison of Raman spectra in Figure 5 shows that, for cells exposed to Ag NPs, there is a distinct concentration dependent increase in the intensity of the 700 cm^−1^ band (which we attribute to C-S vibrations in methionine). Raman spectroscopy has been applied to studies of sulpher-containing amino acids by many groups [69]–[73]. The reported C-S stretching frequency in the mercury-metheonine complex lies in the range 692–714 cm^−1^ [69]. An extensive SERS study of methionine-containing short peptides was reported recently [70] which highlighted the importance of a prominent band in the 660–690 cm^−1^ range which was ascribed to C–S stretching. Another SERS study of methionine has revealed two C-S vibrational frequencies, one at 700 cm^−1^ and the other at 720 cm^−1^ [71]. A prominent Raman peak at 700 cm^−1^, due to C-S stretching of the methionine [72], was also reported by Stewart et al. in their SERS study. The consensus that has been established in the literature on the 700 cm^−1^ line being ascribed to C-S vibrations has encouraged many workers to claim detection of the methionine residue in neurotoxins on the basis of detection of this line (see, for instance, ref. [73]).

There are inconsistent variations in the relative intensities of several other vibrational bands, and we could not find any concrete link with specific biochemical modifications. It has been reported that the exposure of cultured human lung epithelial cells to cerium oxide nanoparticles may lead to an increase in ROS (reactive oxygen species) and cell death, indicating that the mechanism of NP-induced cytotoxicity may be through oxidative stress [74]. Methionine is known to have antioxidant effects that lead to reduction of lipid peroxidation, protection against membrane damage and restoration of changes in the glutathione system [75]. The increase in the intensity of the methionine-related peak (700 cm^−1^) with Ag NP concentration that we observe in Fig. 6 indicates enhanced resistance of mesenchymal stem cells against oxidative stress induced by Ag NPs at concentration levels up to 4 µg/ml. It is pertinent to note here that the concentrations of Ag NPs used in our measurements seem to induce oxidative stress.

**Figure 7:**
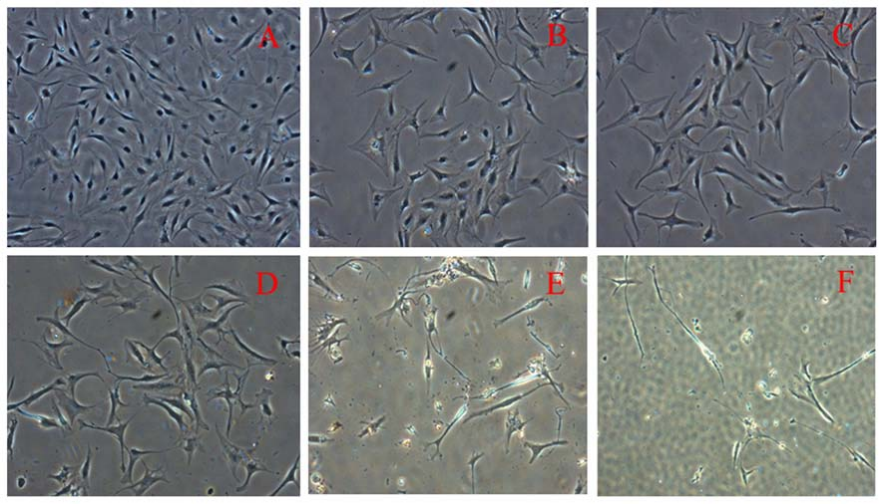
Raman spectrum of Ag NPs recorded using 10 mW power of 785 nm laser beam. Acquisition time: 30 s with 5 average accumulations. The top panel shows the raw spectrum and lower panels shows the spectrum after appropriate background subtraction.

In order to check the spectral contribution from the nano-particles on their own, we have recorded control micro-Raman spectra of film of Ag NPs in the sample cell; Figure 7 shows typical raw and base line corrected spectra. Although it was not possible to record spectra of NPs in solutions of different concentrations (due to extremely small Raman cross sections), the spectrum shown in Fig. 7 clearly establishes the absence of any vibrational line at 700 cm^−1^, suggesting that the stress marker peak at 700 cm^−1^ is not contributed by Ag NPs.

To understand the behavior of the cellular membranes following NP exposure, we studied the ratio of the Raman band intensity of the lipid deformation mode at 1302 cm^−1^ to that of the Amide III band at 1268 cm^−1^ for cells exposed to different concentrations of Ag NPs. Earlier studies have reported that adverse effects of nanoparticle cytotoxicity include damage to cell membranes and to DNA [54], [76], [77] and it has been reported that these ratios are good Raman markers of such changes [67]. The DNA damage in the exposed cells was elucidated by determining the intensity ratio of the 725 cm^−1^ band (corresponding to the vibration of the DNA base Adenine) to that of Amide III band at 1268 cm^−1^. A decrease in these values on increase in toxicity is attributed to cell membrane and DNA damages [67]. In our experiments, the values of Lipid/Amide III ratio obtained for control, AgNP-treated cells with concentrations 1 µg/ml, 2 µg/ml, and 3 µg/ml are 1.85. 1.97, 1.78, 1.72 and 1.62 respectively and the Adenine/Amide III ratios are 0.62, 0.58, 0.57, 0.72 and 0.67, respectively. A closer look at the Lipid/Amide III data indicates membrane damage whereas the values that correspond to Adenine/Amide III ratios are not supportive of the notion that there is DNA damage.

### Statistical Analysis

**Figure 8:**
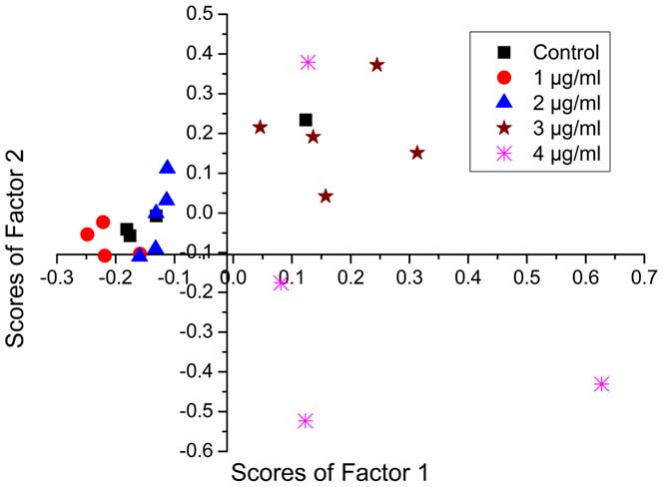
PCA results in the form of a scatter plot depicting the Score of Factor 1 against the Score of Factor 2. Control, 1 µg/mL and 2 µg/mL NP treated cell spectra are clustered together (except one datum).

Principal component analyses [78], [79] of all the spectra that we recorded of control and nanoparticle-treated samples were performed after normalization with respect to the 998 cm^−1^ peak and results are shown in Fig. 8. The figure depicts a plot of scores of the first factor and scores of the second factor that is orthogonal to the first. There is clear demarcation between the two categories of spectra corresponding to the cells treated with NP doping levels of 1–2 µg/ml and cells with higher doping levels (3–4 µg/ml).

We have chosen 6 factors for the PCA analysis. The first factor itself defines the data well with more than 59% variance. It is pertinent to note that our PCA was performed in the low frequency region, spanning the range 440 cm^−1^ to 1015 cm^−1^, where there is no background signal from glass. Moreover, the spectra in this region were background corrected and normalized before loading for PCA analysis. Hence the PCA results we report are exclusive of any effects of (or changes in) background signal. It must also be noted that obtaining better statistics would be desirable but was not practical with the stem cell samples available in the present study.

To summarize, on the basis of the present series of experiments we are able to deduce that at NP doping levels in excess of 2 µg/ml, there exists discernable nanoparticle-induced oxidative stress on hMSC leading to the death of cells at NP concentration levels in excess of 5 µg/ml. While higher doping levels of Ag NPs lead to enhanced antimicrobial effects, it is clear that such doping levels induce larger amounts of stress on human cells. The present work seems to lend support to the notion that the physical and chemical properties of Ag NPs induce oxidative stress on hMSC and that such stress may well be a precursor to cytotoxic effects. A recent study by Greulich et al. [80] has shown concentration-dependent activation of hMSCs at nanosilver levels of 2.5 µg/mL, and cytotoxic cell reactions at Ag NP concentrations above 5 µg/mL. It is, however, important to take cognizance of the fact that proper comparison of toxicity studies involving Ag NPs may be complicated by different nanoparticle sizes, particle form, particle coatings, and different experimental design [81].

It is noteworthy that our results suggest that NP-induced stress occurs independently of whether the Ag NPs are taken in by the cells, or remain on the cell membrane, or, indeed, are in close proximity to the cells. Nevertheless, it will be interesting to conduct electron microscopy studies to probe whether NP intake into the cells is a necessary condition for inducing stress.

It has been well recognized that free radicals are frequently generated upon introduction of diverse nanomaterials, and such free radicals play an important role in generating NP-induced toxicity [8]. Free radicals give rise to oxidative stress, inflammation and consequent damage to proteins, membranes and DNA [82]. Our present Raman spectroscopy analysis has shown an anti-oxidant effect of methionine in NP-doped cells, suggesting that Raman spectroscopy combined with optical tweezers may prove to be an appropriate method to unravel the mechanism leading to the NP-induced toxicity using individual cells kept under physiological conditions. Our results appear to be in consonance with what has been established in a general context in several earlier studies, that whenever a cellular system is under oxidative stress it will produce antioxidants as a protective mechanism [68], [72], [73]. Methionine is found by us to be an important cellular antioxidant.

It would clearly be of interest to apply our trap-based method to probe contemporary issues that are presently explored using conventional biochemical techniques, issues such as how NP size-dependence might affect the generation of reactive oxygen species in cellular materials [83] and how NP-induced physiochemical alterations might be correlated with NP-toxicity [84]–[87].
