# Correction: Micro-Raman Spectroscopy of Silver Nanoparticle Induced Stress on Optically-Trapped Stem Cells

**DOI:** 10.1371/annotation/60f68765-e790-4ab1-b1f2-3867100c6e3e

**Published:** 2012-07-09

**Authors:** Aseefhali Bankapur, R. Sagar Krishnamurthy, Elsa Zachariah, Chidangil Santhosh, Basavaraj Chougule, Bhavishna Praveen, Manna Valiathan, Deepak Mathur

The images for Figures 4, 5, 6, and 7 were incorrectly switched. The image that appears as Figure 4 should be Figure 5, and the image that appears as Figure 5 should be Figure 6. The image that currently appears as Figure 6 should be Figure 7, and the image that appears as Figure 7 should be Figure 4. 

